# Circumcision‐Associated Penile Papules: A Novel Complication With Unclear Etiology

**DOI:** 10.1111/jocd.70034

**Published:** 2025-02-12

**Authors:** Mingshuang Zhang, Rongqing Yang, Jie Huang

**Affiliations:** ^1^ Department of Dermatology Shenzhen Third People's Hospital Shenzhen Guangdong China

**Keywords:** case report, circumcision, complications, genital warts, penile, post‐circumcision papules

## Abstract

**Objective:**

To report six patients with penile papules after circumcision to attract the attention of clinicians and patients to the complication associated with circumcision.

**Methods:**

We reported several clinical cases presenting with these papules following circumcision. Cases were reassessed for clinical characteristics, histopathological features, and potential risk factors.

**Results:**

A series of six adult male cases were identified, with papules appearing asymptomatically months to years after circumcision. The papules were larger, exhibited uneven distribution, and lacked the typical arrangement of pearly penile papules, posing a cosmetic concern and a risk of misdiagnosis as genital warts.

**Conclusion:**

This study presents a previously unreported complication of circumcision with significant implications for patient care. Further investigation is warranted to understand the etiology and develop strategies for prevention and management.

## 1 Introduction

Circumcision is one of the most frequently performed surgical procedures worldwide, with an estimated 200 million males undergoing the procedure annually [1]. It is particularly prevalent in certain religious and cultural groups, with rates exceeding 90% in the Middle East and North Africa [2]. Although circumcision is generally considered safe and beneficial, it is not without potential complications. As with any surgical procedure, complications after a male circumcision surgery are possible. Postoperative complications can range from minor issues like bleeding and pain to more severe conditions such as balanitis, phimosis, and urethral stricture [3]. Some of these complications are minor and easily treated such as bleeding (in patients without a bleeding disorder) and infection. Others, however, require additional surgery to correct the complication such as trapped penis and unsatisfactory cosmetic results [4, 5]. Some complications are irreversible such as decreased sexual sensation and death. Besides, psychological issues have been reported to arise in children after operations, including circumcisions [6].

In recent years, a novel complication associated with circumcision has emerged: penile papules [7, 8, 9, 10]. They are small, painless, skin‐colored papules that typically appear along the coronal sulcus and frenulum of the penis months or years after circumcision [11, 12]. Despite their benign nature, post‐circumcision papules (PCPs) can cause significant cosmetic concerns and may be misdiagnosed as genital warts, leading to psychological distress and social impairment. However, clinicians still have insufficient understanding of the clinical manifestations, etiology, and treatment of post‐circumcision penile papules.

Thus, the purpose of this study was to report six patients with post‐circumcision penile papules to inform clinicians and patients about the complication associated with circumcision procedures. The study also explored the relationship between surgical methods and the occurrence of post‐circumcision penile papules. And the informed consents have been obtained from all the patients in this case reports.

## 2 Case Reports

A retrospective review was conducted on cases that presented to our department in recent years with papules on the penile shaft and coronal sulcus following circumcision. Each case was reassessed and interviewed in detail regarding preoperative characteristics, surgical procedures, postoperative management, and follow‐up. Additionally, histopathological examinations of the local tumors were performed for typical cases. We present six cases in adult male patients who underwent circumcision. The patients were aged between 28 and 32 years old and presented with small, painless, skin‐colored papules along the coronal sulcus and frenulum of the penis. The papules ranged in size from 2 to 5 mm and were typically asymptomatic. The duration of symptoms ranged from 1 months to 10 years after circumcision. The detailed characteristics of six patients were shown in Table 1. In addition, we also presented three cases with typical PCPs as follows. This study was performed in line with the principles of the Declaration of Helsinki. The Ethics Review Committee of our hospital, approved this study (Ethical approval number: 2022‐102).

**TABLE 1:** The detailed characteristics of six patients with post‐circumcision papules.

Patient	Age (years)	Duration of symptoms	Papule size (mm)	Papule location	Pathology	HPV	Treatment	Outcome
1	28	10 years	3–4	Coronal sulcus, surgical incision site	Squamous epithelial hyperplasia	Negative	Cryotherapy	Improvement
2	30	8 months	3	Coronal sulcus, surgical incision site	Polypoid changes covered by squamous epithelium	Positive	Cryotherapy	Recovery
3	32	1 month	3–4	Coronal sulcus, frenulum	Squamous epithelial hyperplasia	Negative	Laser ablation	Improvement
4	29	3 months	3–4	Coronal sulcus, frenulum	Squamous epithelial hyperplasia	Negative	Laser ablation/Surgical excision	Resolution
5	31	2 years	3–4	Coronal sulcus, frenulum	Squamous epithelial hyperplasia	Negative	Surgical excision	Resolution
6	31	1 month	3	Coronal sulcus, frenulum	Squamous epithelial hyperplasia	Negative	Surgical excision	Resolution

### 2.1 Case 1

**FIGURE 1:**
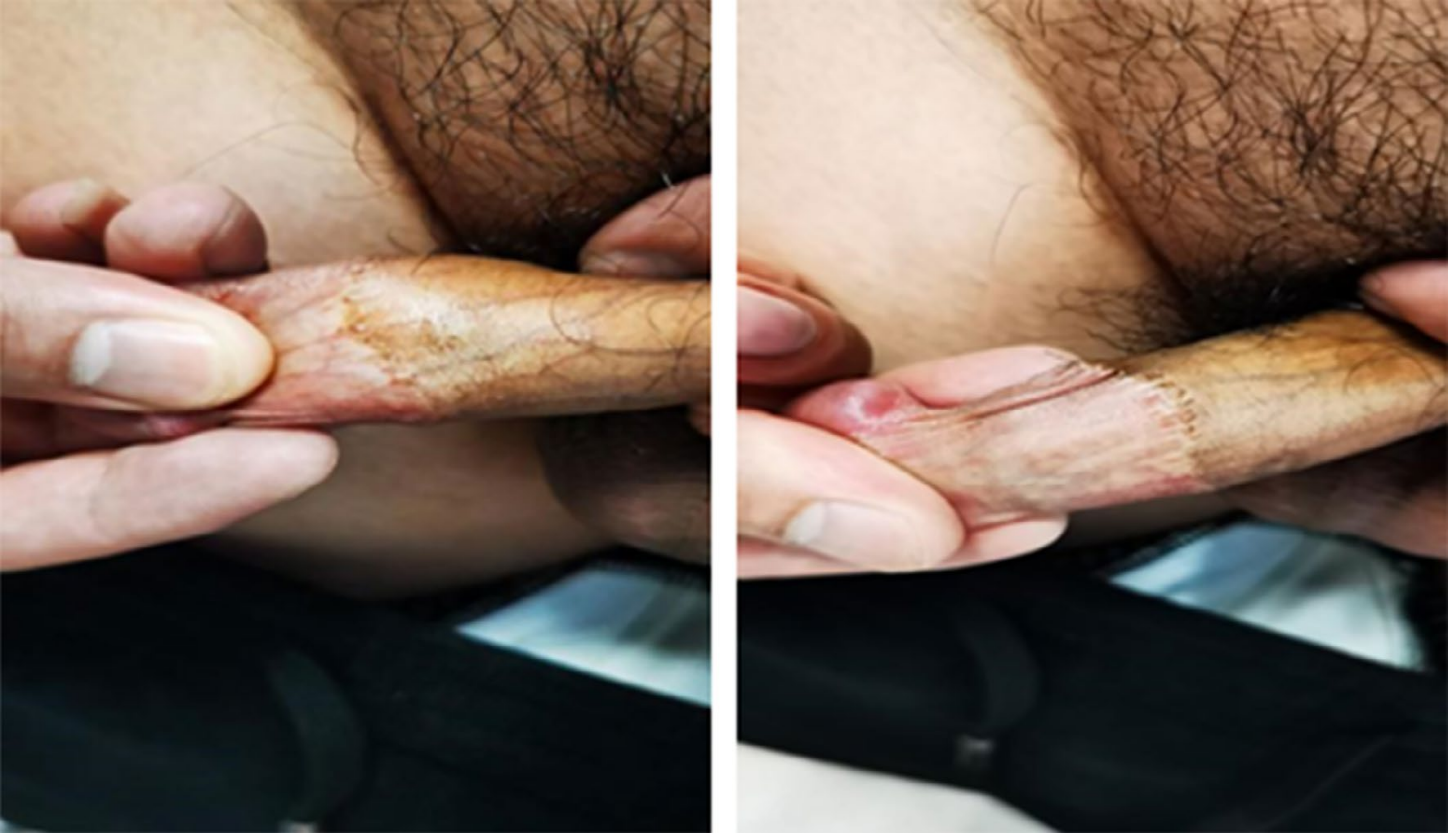
The physical examination of Case 1. Intense, millet‐sized protrusions were observed along the coronal sulcus of the external genitalia, totaling more than 20 nodules, and no tenderness upon palpation was observed.

**FIGURE 2:**
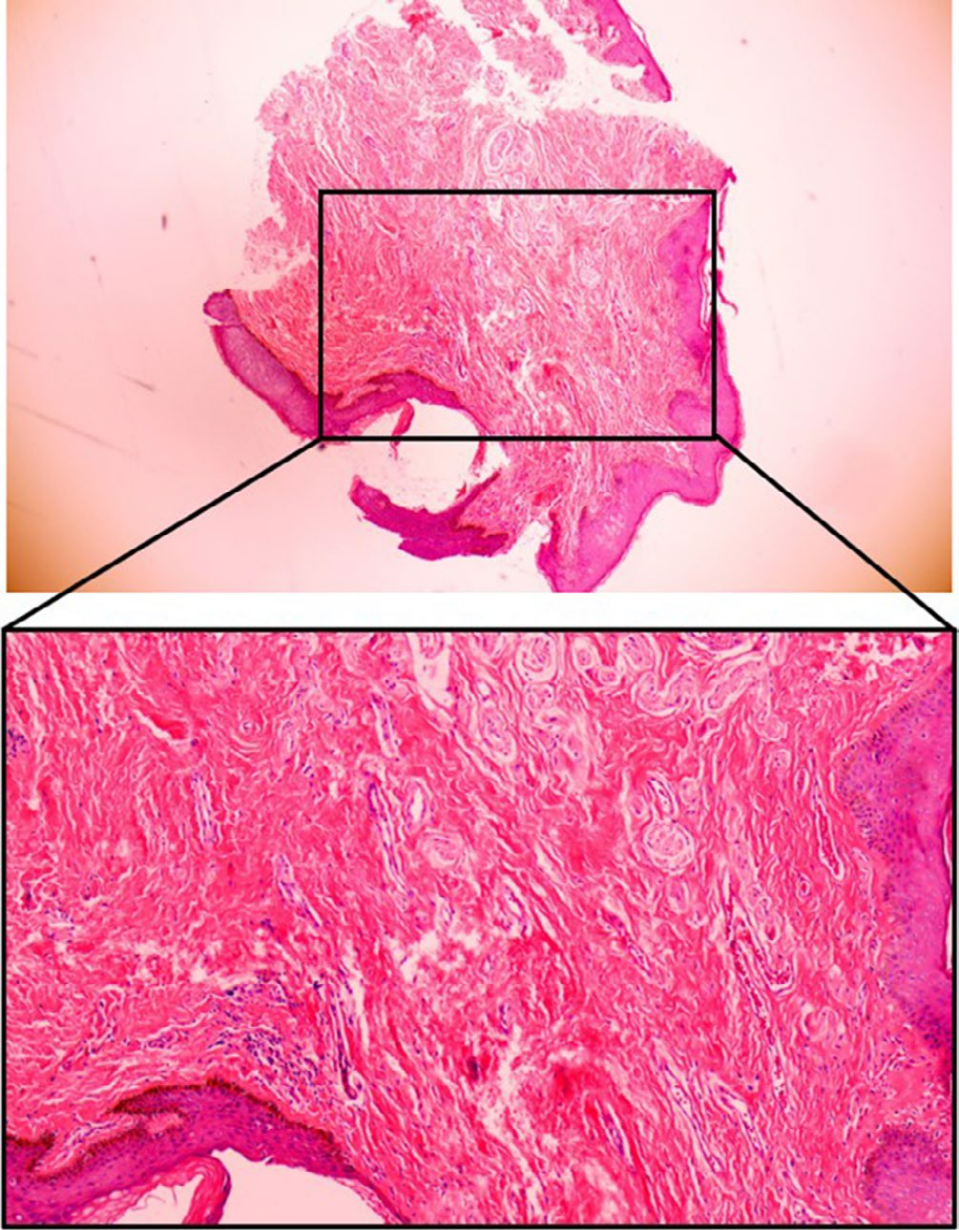
Pathologic results of case 1 (H&E staining).

A 28 years old male patient was admitted to our hospital with intense, millet‐sized protrusions along the coronal sulcus of the penis for 10 years. The patient underwent circumcision for an excessively long prepuce 10 years ago. Two months post‐surgery, he noticed the emergence of millet‐sized flesh‐colored protrusions at the incision site, which gradually increased in number. These protrusions were painless and itchless. Recently, his partner was diagnosed with HPV infection, prompting the patient's concern about sexually transmitted diseases and seeking medical examination. He has no similar history and other disease history. The physical examination showed that intense, millet‐sized protrusions were observed along the coronal sulcus of the external genitalia, totaling more than 20 nodules, and no tenderness upon palpation was observed (Figure 1). Clamp sampling and pathological examination of the lesion site showed that human papillomavirus (HPV) was negative, and the final diagnosis was considered as PCPs (Figure 2).

### 2.2 Case 2

**FIGURE 3:**
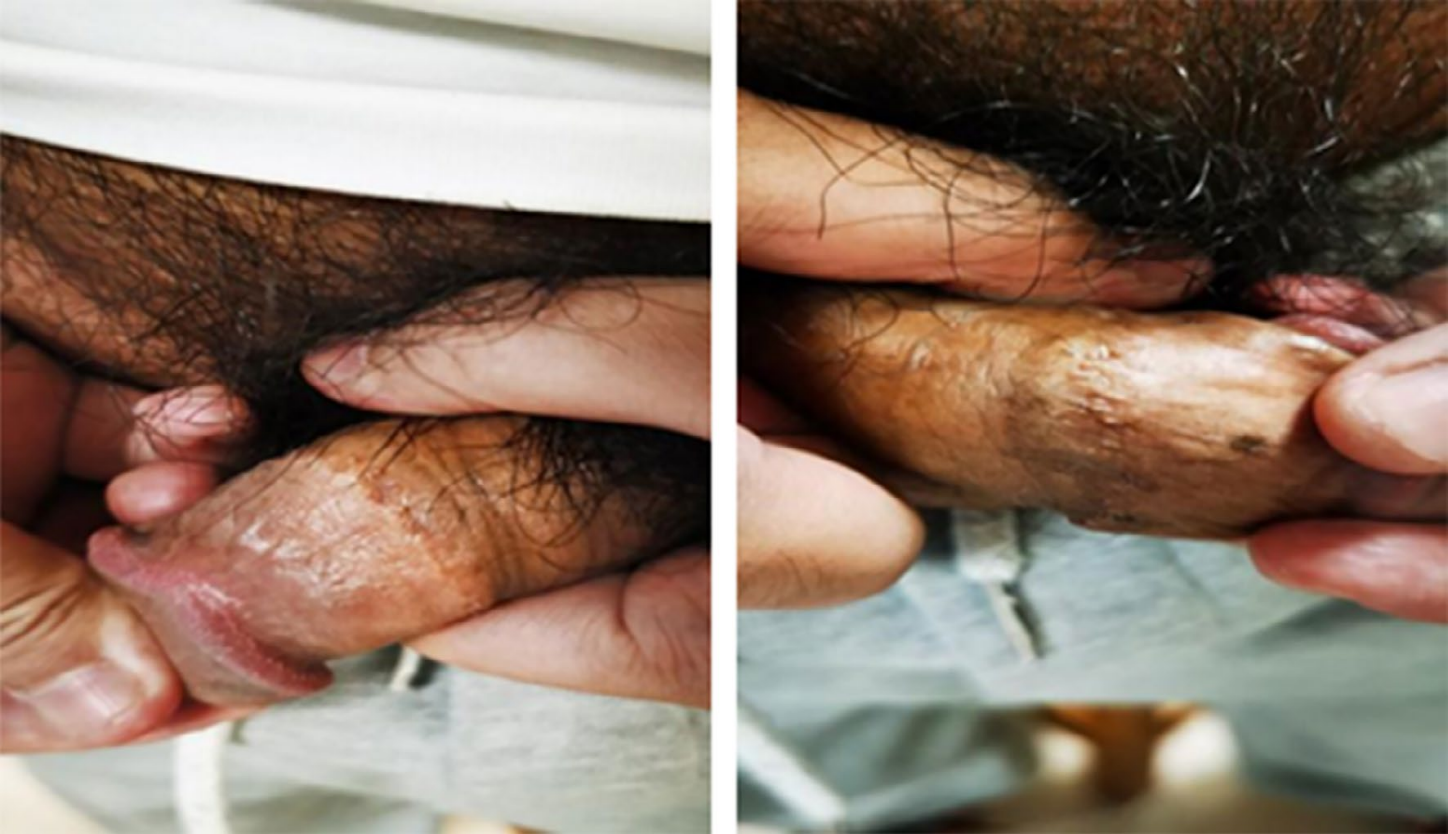
The physical examination of Case 2. The lesions in the mentioned area had regressed after cryotherapy, and small nodules are present at the circumcision site.

**FIGURE 4:**
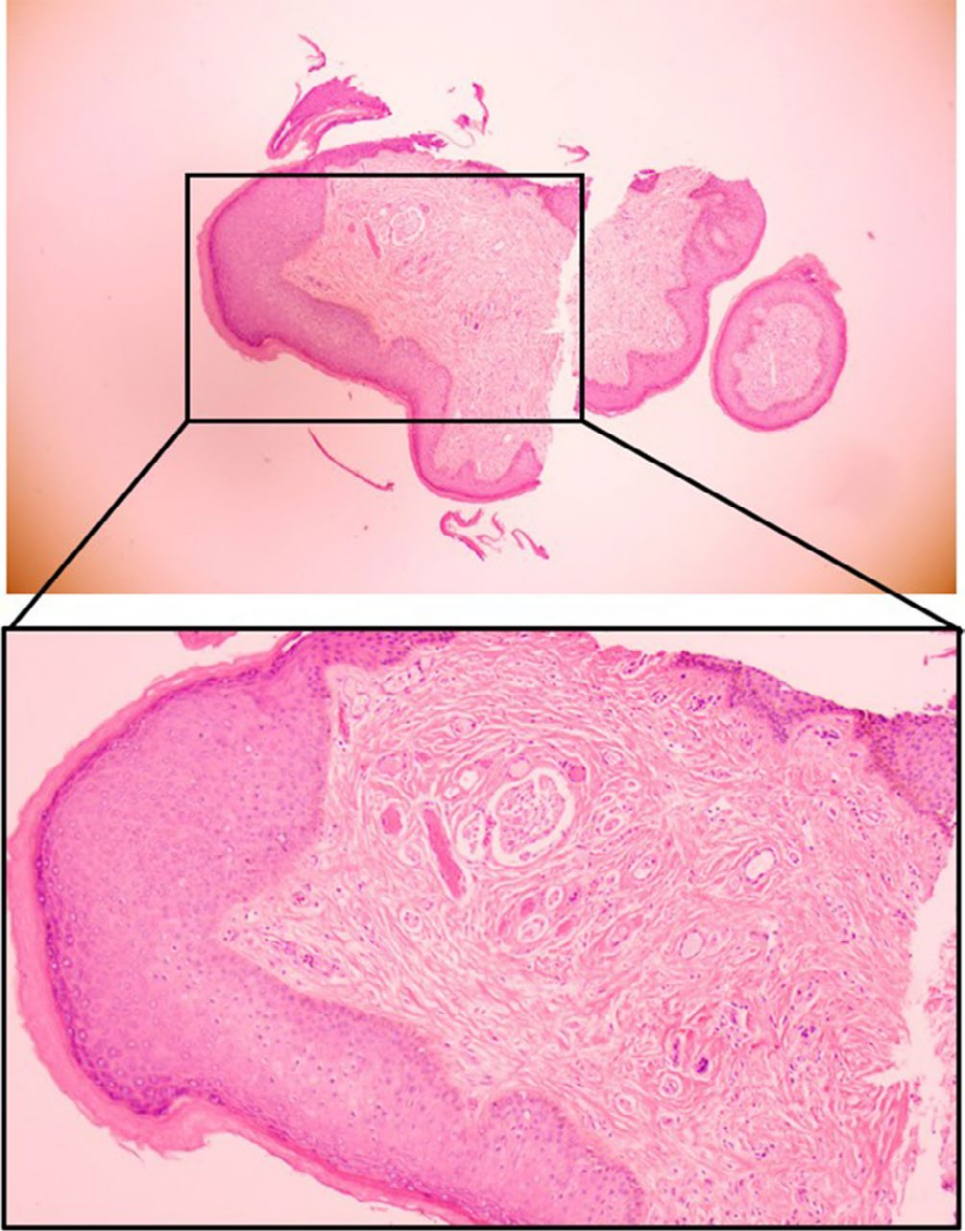
Pathologic results of case 2 (H&E staining).

A 30‐year old male patient was admitted to our hospital with detection of penile lesions in the coronal sulcus for 8 months. The patient presented with penile lesions in the coronal sulcus that appeared without an obvious cause 8 months ago. He received laser and cryotherapy treatments once at another hospital. One month after cryotherapy, the lesions were not detected upon review. In April 2022, he underwent a circumcision with staple suturing, and small nodules appeared at the circumcision site post‐surgery. He has no similar history and other disease history. The physical examination showed that the lesions in the mentioned area had regressed after cryotherapy, and small nodules are present at the circumcision site (Figure 3). The T‐lymphocyte subset examination (December 22, 2023) showed: Helper T lymphocytes (CD3^+^CD4^+^) 23.0%, Helper T lymphocytes absolute count (CD3^+^CD4^+^) 548 cells/ul, Suppressor T lymphocytes (CD3^+^CD8^+^) 43.8%, Helper T/Suppressor T ratio (CD3^+^CD4^+^/CD3^+^CD8^+^) 0.52. Complement 3 detection (December 22, 2023) showed: 1.576 g/L. The result of HPV test at coronal sulcus (February 6, 2024) was negative. The pathological diagnosis was considered as PCPs (Figure 4).

### 2.3 Case 3

**FIGURE 5:**
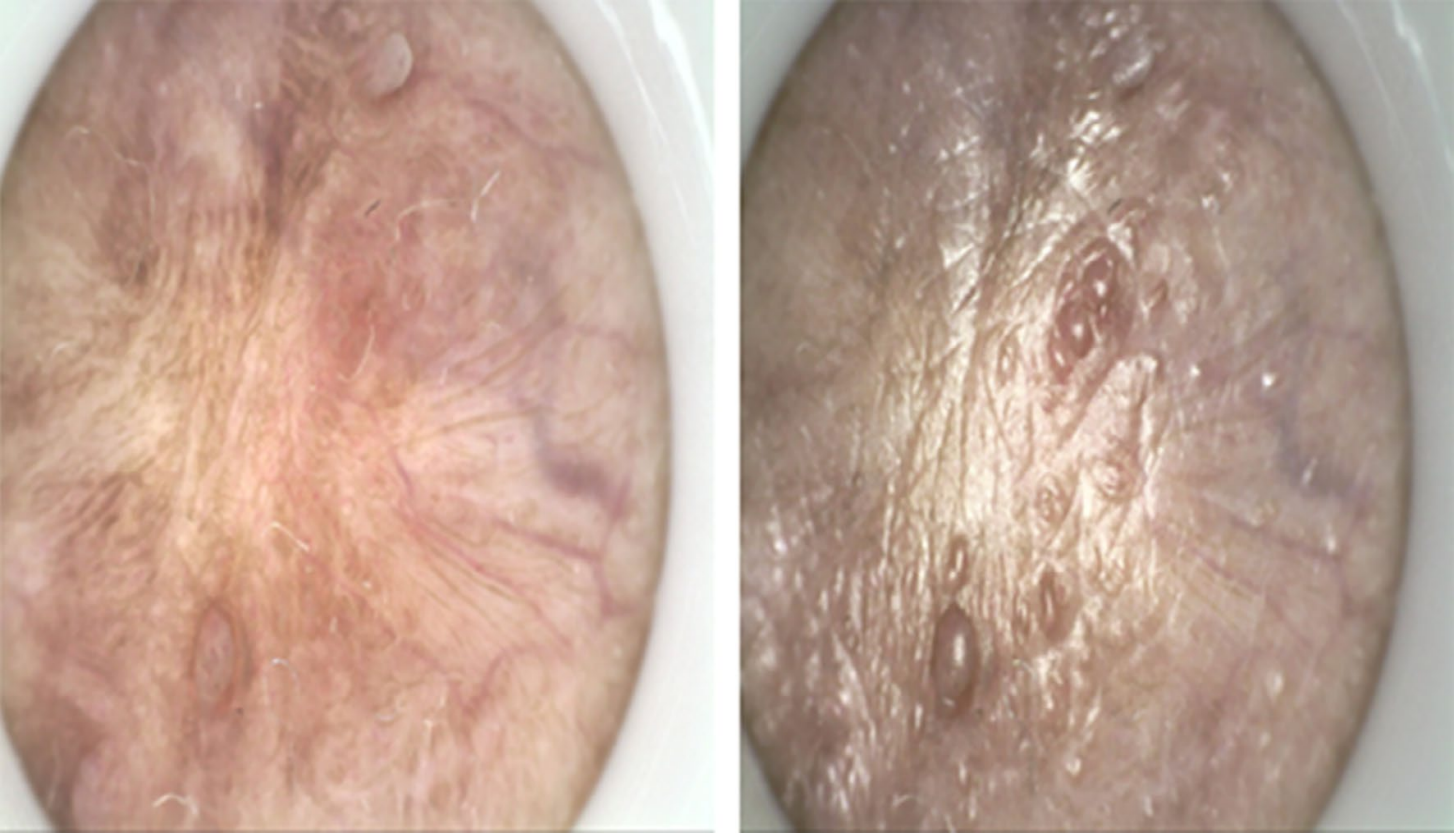
The physical examination of Case 3 via dermatoscope. Several millet‐sized, flesh‐colored protrusions with smooth surfaces were visible.

**FIGURE 6:**
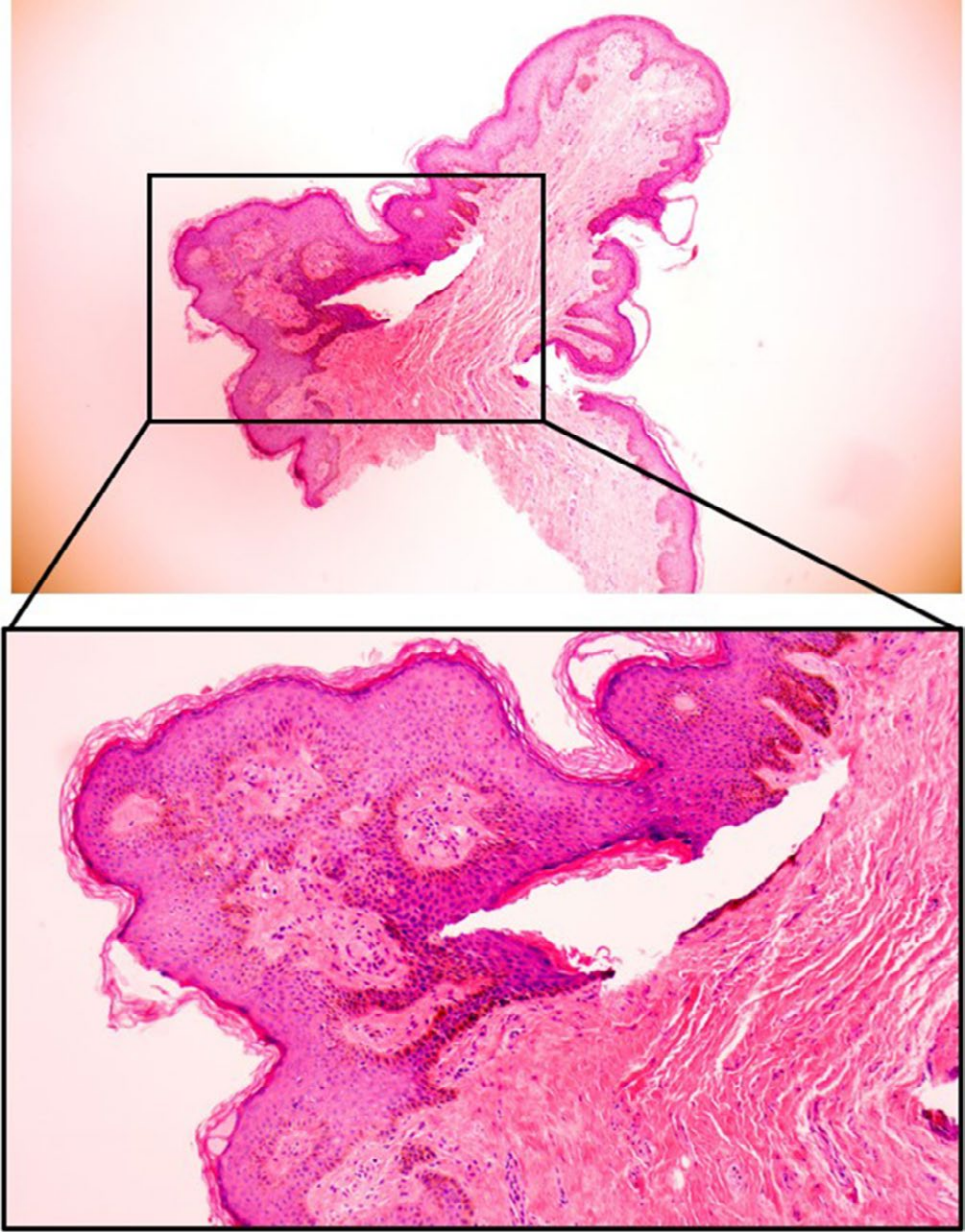
Pathologic results of Case 3 (H&E staining).

A 32 years old male patient was admitted to our hospital with small bumps discovered at the circumcision incision site a months ago. The patient underwent circumcision 20 years ago. Recently, without any apparent cause, he started experiencing the aforementioned symptoms, which are neither painful nor itchy. Six months ago, the patient received laser treatment for sharp, moist warts at the pubic mound and applied interferon gel topically. He has no similar history and other disease history. The physical examination showed that several millet‐sized, flesh‐colored protrusions with smooth surfaces were visible (Figure 5). The pathological examination of penile skin tumor (January 15, 2024) showed that the tissue presented as polypoid changes, covered by squamous epithelium with papillary hyperplasia, slight infiltration of lymphocytes in the stroma, no evident koilocytosis and atypical cells were observed (Figure 6). Besides, HPV test showed negative. The diagnosis was considered as PCPs.

## 3 Discussion

The emergence of PCPs several years after the surgical procedure presents a complex clinical scenario, suggesting a multifactorial etiology involving surgical technique, postoperative care, and individual patient factors [7, 13]. The delayed presentation of these papules resembles other long‐term complications such as skin bridges, indicating a possible connection in their pathophysiological development [14].

During the surgical process, if the operations such as cutting and suturing of the foreskin and surrounding tissues are not performed precisely enough, it may lead to excessive damage to the local tissues [2, 3]. For example, when using a traditional surgical scalpel, an irregular incision and incomplete hemostasis may occur. Or when using the stapler method, improper squeezing and cutting forces of the instrument on the tissues can cause ischemia and necrosis of the tissues, all of which will trigger an excessive repair response of the body, promote the proliferation of fibrous tissues, and consequently form papules. If too little foreskin is removed, the remaining inner plate tissue may experience abnormal proliferation of epithelial cells due to factors such as changes in the local environment and friction, thus forming papules. On the other hand, if too much foreskin is removed, it may result in an overly tight fit between the glans and the surrounding skin, increasing the friction during daily activities, and long‐term stimulation can also easily trigger the formation of papules [2, 3].

In addition, improper wound care after the surgery is prone to cause local infections. The invasion of pathogens such as bacteria and viruses will trigger an inflammatory response [1]. The inflammation stimulates the surrounding tissues, leading to cell proliferation and tissue edema. Long‐term chronic inflammation can cause the local tissues to repeatedly repair and proliferate, ultimately forming papules. For instance, *Staphylococcus aureus* infection can cause local suppurative inflammation, and papule‐like changes may remain after healing [13]. Furthermore, during the wound healing process, excessive proliferation of fibrous tissues forms scars. Scar tissues have poor elasticity and differ from the surrounding normal tissues in structure and function. They are easily subjected to stimuli such as friction and traction, which in turn can trigger abnormal reactions of the local tissues and produce papules. Moreover, scar contracture may also change the distribution of skin tension in the local area, further promoting the formation of papules [2, 3]. In addition, the PCPs may arise due to chronic mechanical irritation from clothing or sexual activity, exacerbated by the formation of scar tissue which is less elastic and more prone to injury over time [15].

Although the role of viral infections in the development of PCPs has not been directly proven, it cannot be completely ignored [16]. Several patients in our study had a history of local infections after circumcision, suggesting that inflammatory responses to these infections could contribute to the development of these papules. Inflammation can lead to local tissue changes, including hyperplasia and abnormal healing, which might predispose to the formation of PCPs. Moreover, the histopathological analysis did not reveal typical signs of viral infections such as koilocytosis, but the persistent inflammation could still play a contributory role.

Furthermore, the majority of patients with PCPs had undergone newer circumcision techniques, such as the stapler method, which has been associated with a higher incidence of local complications including infection [13]. The stapler method, although efficient, may create more tissue trauma and necrosis due to the mechanical nature of the device, potentially leading to increased inflammatory responses and abnormal wound healing processes. This, combined with the observed higher incidence of postoperative infections, suggests a potential link between these surgical methods and the development of PCPs.

Given these observations, it is imperative to conduct further research to clarify the exact mechanisms by which these factors contribute to the development of PCPs. Such studies should aim to explore the relationship between different circumcision techniques and postoperative care protocols, with a particular focus on minimizing tissue trauma and optimizing healing. Preventive measures could include meticulous surgical techniques to reduce skin trauma, enhanced infection control protocols, and perhaps the use of anti‐inflammatory agents postoperatively to minimize inflammatory responses.

Taken together, the development of PCPs seems to be a multifaceted issue influenced by mechanical irritation, surgical techniques, inflammatory processes, and possibly latent infections. A deeper understanding of these interrelated factors will help develop targeted interventions to prevent this complication, thereby improving patient outcomes after circumcision.

Nevertheless, this study still has some limitations. Firstly, this study only retrospectively reported several cases of PCPs patients, and the sample size was not large enough. Therefore, it was impossible to conduct a systematic analysis to explore the causes of the formation of PCPs. Secondly, we did not provide detailed and comprehensive background information of the patients, such as lifestyle habits, sexual history, and past medical history. And these pieces of information might be related to the occurrence and development of penile papules associated with circumcision (PCPs). Thirdly, we did not explore the impact of PCPs on the quality of life of patients. Therefore, in subsequent studies, we will further increase the sample size and collect more clinical and pathological data of patients to evaluate the influencing factors of the formation of PCPs.

## 4 Conclusion

PCPs constitute an under‐recognized post‐circumcision complication with significant implications for patient care and satisfaction. Further investigation is required to unravel the etiology and risk factors associated with PCPs. It is imperative for clinicians to be aware of the distinct features of PCPs to prevent misdiagnosis and to provide appropriate patient reassurance and management. Future research should concentrate on developing preventive strategies, informed by a deeper understanding of the underlying mechanisms, to reduce the incidence of PCPs and enhance post‐circumcision patient outcomes.

## Author Contributions

M.Z. contributed to the guarantor of integrity of the entire study, study concepts, definition of intellectual content, literature research, clinical studies, data acquisition and analysis, statistical analysis; J.H. contributed to study design, literature research, clinical studies, manuscript preparation, and editing and review; R.Y. contributed to the clinical studies, data acquisition. All authors read and approved the final manuscript.

## Ethics Statement

This study was performed in line with the principles of the Declaration of Helsinki. The Ethics Review Committee of Shenzhen Third People's Hospital (Ethical approval number: Shenzhen Third Institute Lunshen Research Project Approval Letter [2022‐102]), approved this study.

## Consent

The authors have nothing to report.

## Conflicts of Interest

The authors declare no conflicts of interest.

## Data Availability

The data that support the findings of this study are available from the corresponding author upon reasonable request.
